# Risk factors for mortality in elderly and very elderly critically ill patients with sepsis: a prospective, observational, multicenter cohort study

**DOI:** 10.1186/s13613-019-0495-x

**Published:** 2019-02-04

**Authors:** Ignacio Martin-Loeches, Maria Consuelo Guia, Maria Sole Vallecoccia, David Suarez, Mercedes Ibarz, Marian Irazabal, Ricard Ferrer, Antonio Artigas

**Affiliations:** 1 Multidisciplinary Intensive Care Research Organization (MICRO), St James’s Hospital/Trinity College Dublin TCD, James’s St, Ushers, Dublin, D03 VX82 Ireland; 2 Pulmonary Intensive Care Unit, Respiratory Institute, Hospital Clinic of Barcelona, IDIBAPS, Barcelona, Spain; 3 University of Barcelona, Barcelona, Spain; 4 Centro de Investigación Biomédica en Red-Enfermedades Respiratorias (CIBERES CB06/06/0028), Barcelona, Spain; 5 Critical care Center, Corporacion Sanitaria Universitaria Parc Tauli, CIBER Enfermedades Respiratorias, Autonomous University of Barcelona, Sabadell, Spain; 6 Department of Intensive Care and Anaesthesiology, Università Cattolica del Sacro Cuore - Fondazione Policlinico Universitario A.Gemelli, Rome, Italy; 7 Servicio de Medicina Intensiva, Hospitales Universitarios Sagrado Corazon y General de Cataluña, Barcelona, Spain; 8 Intensive Care Department, Vall d’Hebron University Hospital, Shock Organ Dysfunction and Resuscitation Research Group, Vall d’ Hebron Research Institute, Passeig de la Vall d’Hebron, 119-129, 08035 Barcelona, Spain

**Keywords:** Elderly, Aging, ICU, Sepsis, Septic shock, Bundles, Critical care

## Abstract

**Background:**

Age has been traditionally considered a risk factor for mortality in elderly patients admitted to intensive care units. The aim of this prospective, observational, multicenter cohort study is to determine the risk factors for mortality in elderly and very elderly critically ill patients with sepsis.

**Results:**

A total of 1490 patients with ≥ 65 years of age were included in the study; most of them 1231 (82.6%) had a cardiovascular failure. The mean age (± SD) was 74.5 (± 5.6) years, and 876 (58.8%) were male. The patients were divided into two cohorts: (1) elderly: 65–79 years and (2) very elderly: ≥ 80 years. The overall hospital mortality was 48.8% (*n* = 727) and was significantly higher in very elderly compared to elderly patients (54.2% vs. 47.4%; *p* = 0.02). Factors independently associated with mortality were APACHE II score of the disease, patient location at sepsis diagnosis, development of acute kidney injury, and thrombocytopenia in the group of elderly patients. On the other hand, in the group of very elderly patients, predictors of hospital mortality were age, APACHE II score, and prompt adherence of the resuscitation bundle.

**Conclusion:**

This prospective multicenter study found that patients aged 80 or over had higher hospital mortality compared to patients between 65 and 79 years. Age was found to be an independent risk factor only in the very elderly group, and prompt therapy provided within the first 6 h of resuscitation was associated with a reduction in hospital mortality in the very elderly patients.

## Introduction

The increase in life expectancy in developed countries has led to greater demand for the admission of the elderly patients in hospital and in intensive care units (ICU). At least, more than half of the patients admitted to ICU are older than 65 years [1–3].

Sepsis is defined as life-threatening organ dysfunction caused by a dysregulated host response to infection [4, 5]. It accounts for more of 20% of ICU admissions with increasing severity over the last years [6]. Septic shock is a subcategory of sepsis associated with a greater risk of mortality than sepsis alone [7].

The incidence of sepsis increases with age, causing a sharp incidence in people older than 80 years, and is associated with extremely high mortality rates [8, 9]. A decade ago, Martin et al. showed that in patients admitted with sepsis, age was an independent predictor of mortality [8, 10–12]. For this reason, ICU physicians are, in general, reluctant to consider ICU admission to elderly patients, despite the presence of clinical criteria indicating that is appropriate [13].

Recent studies are showing that age is not a critical determinant risk factor for survival in elderly patients. A recent study that included 2646 patients, with a median age of 87 years, found that predictors of in-hospital death were more related to immediate severity conditions (severity score, condition potentially warranting ICU admission, and decubitus ulcers) than the age itself [14]. This hypothesis was confirmed by another study involving over 5000 patients older than 80 years (VIP1 study), where it was demonstrated that age had a smaller impact on survival in ICU and other factors could predict better the risk of mortality among these patients [15]. This study included patients with elective and acute admission and, unsurprisingly, patients admitted acutely had more organ failure and higher mortality. For this category of patients, the evidence available still does not help the physicians to decide if less intense settings could guarantee similar or better outcome [16].

There is no clear definition for elderly for patients admitted to an ICU. In this manuscript and following the World Health Organization (WHO) and the Organisation for Economic Co-operation and Development (OECD), “elderly” was considered for an age frame of 65 years or more [1, 2]. The aim of this manuscript was to determine risk factors for mortality in elderly and very elderly critically ill patients with sepsis.

## Methods

### Study design and data source

We analyzed all the patients with 65 years or over enrolled in the multicenter Edusepsis Study group. Age was determined in two categories based on the most often referred term to “elderly” as those aged 65–79. A further analysis was conducted in very elderly patients over 80 years. Patients ≥ 65 years (including very elderly) are referred to “patients aged 65 years or older."

Edusepsis was a prospective, observational cohort study conducted across 77 ICU in Spain using a before-and-after design to evaluate an educational program for patients with sepsis. The before period (pre-intervention) consisted of all consecutive patients with sepsis who were admitted to the participating ICUs 2 months before the educational program began. The intervention was introduced over a 2-month period, during which no patient data were collected. The post-intervention period consisted of all consecutive patients with sepsis (including organ dysfunction) admitted to the participating ICUs during a 4-month period. In addition, to determine the longevity of the effects of the educational program, a third observation period, composed of all consecutive patients admitted to a subset of the participating ICUs during a 2-month period 1 year later, was included [17]. Briefly, the general coordinating center was located at the Department of Intensive Care Medicine of the Parc Tauli/Hospital Sabadell, Barcelona. All participating ICUs were medical–surgical; most of them (68%) were university hospitals with residency training. Compliance was defined as national educational program based on the SSC guidelines could improve compliance with recommended processes of care in sepsis in Spanish ICUs.

### Patients

All ICU admissions from the emergency department or medical and surgical wards and all ICU patients were actively screened daily for the presence of sepsis, which included definitions of sepsis and organ dysfunction. The onset of sepsis (time zero) was determined according to the patient's location within the hospital when sepsis was diagnosed. In patients diagnosed with sepsis in the emergency department, time zero was defined as the time of triage. For patients admitted to the ICU from the medical and surgical wards or other nonemergency department units, time zero was determined by searching the clinical documentation for the time of diagnosis of sepsis. This might include, for example, a physician's note or timed and dated orders, a timed and dated note of a nurse's discussion of sepsis with a physician, or timed records initiating the referral to the ICU for sepsis. If no time and date could be found by searching the chart, the default time of presentation was the time of admission to the ICU. Lastly, for patients who developed sepsis after admission to the ICU, the time of presentation was again determined on the basis of the clinical documentation.

Sepsis was defined as the combination of a known or suspected infection and acute organ dysfunction: (1) respiratory dysfunction, bilateral pulmonary infiltrates with a ratio of PaO2 to FiO2 of less than 300 mm Hg; the worst PaO2/FiO2 at the time of diagnosis of sepsis was introduced entry in the database during the first 24 h after ICU admission was recorded; (2) acute kidney injury (AKI), urine output of less than 0.5 mL/kg per hour for at least 2 h or a serum creatinine level greater of 2.0 mg/dL (150 μmol/L); (3) coagulation abnormalities, international normalized ratio greater than 1.5 or a partial thromboplastin time greater than 60 s; (4) thrombocytopenia, platelet count of less than 100 × 103/μL; (5) hyperbilirubinemia, total plasma bilirubin level greater of 2.0 mg/dL (150 μmol/L); (6) hypoperfusion, lactate level greater than lactate level of 2 mmol/L (18.2 mg/dL); or (7) hypotension, systolic blood pressure < 90 mm Hg, mean arterial pressure (MAP) < 65 mm Hg, or a reduction in systolic blood pressure of greater than 40 mm Hg from baseline. Septic shock was defined as acute circulatory failure (systolic blood pressure < 90 mm Hg, MAP < 65 mm Hg, or a reduction in systolic blood pressure > 40 mm Hg from baseline) despite adequate volume resuscitation.

### Data collection

We recorded demographic and clinical characteristics of all patients included in the study. Age, sex, admission category (medical, surgical and trauma), source of infection (pneumonia, urinary tract infection, abdominal infection, skin and soft tissue infections, catheter-associated bloodstream infection, other infections, two or more infections), and patient location at the time of diagnosis of sepsis (emergency department, medical or surgical ward, ICU) were collected at the time of presentation of sepsis. The level of severity at admission was assessed by a modified [18] Acute Physiology Chronic Health Evaluation II (APACHE II) score (in which the impact of age on the APACHE score was eliminated) and the number of organ failures.

The initial treatment strategy was assessed applying the treatments recommended by the Surviving Sepsis Campaign (SSC) guidelines [19] in the first 24 h after the diagnosis of sepsis, divided into two variables: (1) implementation of all measures of resuscitation in the first 6 h of sepsis (lactate measurement, collection of cultures before starting an antibiotic treatment, administration of broad spectrum antibiotics, administration of fluids and vasopressors to achieve a systolic blood pressure > 90 mmHg or a MAP > 65 mmHg, central venous pressure (CVP) equal to or greater than 8 mmHg, central venous saturation greater than or equal to 70%) and (2) implementation of all measures of treatment within 24 h (including consideration of low doses corticosteroids in patients with septic shock, blood glucose control and control of the plateau pressure for protective mechanical ventilation). The primary outcome of this study was hospital mortality. Secondary outcomes were ICU mortality, 28-day mortality, hospital, ICU length of stay (LOS), and bundle compliance variables. Bundle compliance variables included ten tasks grouped in the sepsis resuscitation bundle (six tasks that should begin immediately and be accomplished within the first 6 h of presentation) and the sepsis management bundle (four tasks that should begin immediately and be completed within 24 h of presentation). Time (0 to 12 h) was also recorded from sepsis presentation to the process of care variables of serum lactate measurement, blood culture collection, administration of broad spectrum antibiotics, achievement of CVP of 8 mm Hg or greater, and central venous oxygen saturation of 70% or greater. As in the Edusepsis study, we controlled the quality of the data gathered, checking for completeness, accuracy, and uniformity. Also, a random sample of 10% of patients was re-evaluated and reliability of 96.5% of all variables per case report form was observed. Each participating centers’ research and ethical review boards approved the study.

### Statistical analysis

Sample size calculation was not performed since this is a prospective observational study conducted during a limited time frame. Because the missing data rate in the study was low (the variable with the highest missing rate was an APACHE II score with a rate of 1.5%), no imputation of missing data was performed. Statistical tests were two-tailed, and significance was set at a level of 0.05. We performed a descriptive analysis of the sample by comparing the two age groups: (1) elderly: 65–79 years of age and (2) very elderly: ≥ 80 years of age. Categorical variables are expressed as frequencies and percentages and analyzed with the Chi-squared test or Fisher exact test, as appropriate. Continuous variables are expressed as means and standard deviations (SD) and compared using the Student’s *t* test. A *p*-value < 0.05 was considered statistically significant. Multivariate logistic regression analysis was performed to assess the impact of age on hospital mortality after adjusting the same models for the two populations: 65–79 years and ≥ 80 years. Variables with *p* < 0.1 in the univariate analysis or clinically relevant were incorporated as follows: Age, APACHE modified score, and ICU LOS were included as continuous variables. Other covariates included were sex, patient location at sepsis diagnosis (emergency department, ward, ICU), type of infection (pneumonia, abdominal, urinary tract, skin and soft tissue, catheter-related bacteraemia, other infections, two or more infections), hemodynamic failure, respiratory failure, AKI, hepatic failure, thrombocytopenia, coagulopathy, resuscitation, and implementation of resuscitation and treatment bundles. To account for center effects in this multicenter trial with a binary outcome, we fitted a generalized estimating equation model with a *logit link* and an exchangeable correlation structure.

## Results

### Patient characteristics

**Fig. 1:**
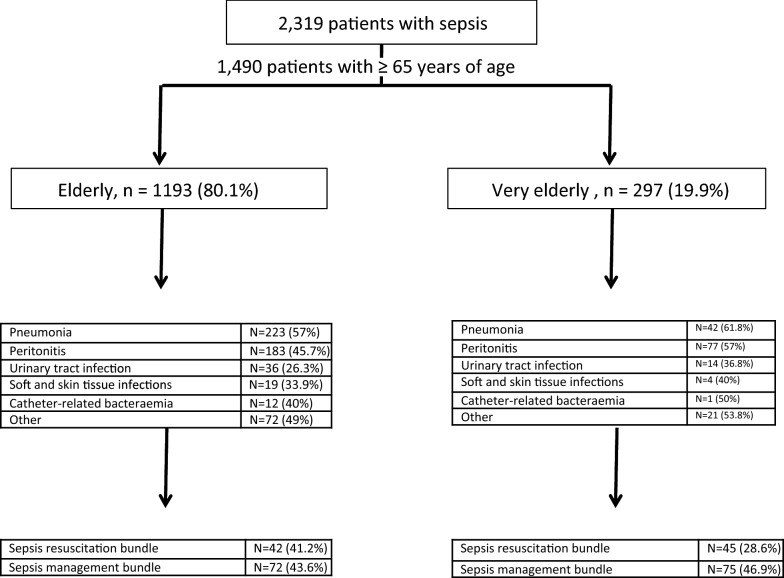
Flow chart for the study

A total of 1490 patients with ≥ 65 years of age were included in the study; most of them 1231 (82.6%) had a cardiovascular failure. The mean age (± SD) was 74.5 (± 5.6) years, and 876 (58.8%) were male. The mean APACHE II score and modified APACHE II score were 22.6 (± 7.0) and 17.1 (± 7.0) points, respectively. Compliance with the SSC recommendations was 7.8% for the sepsis resuscitation bundle (6 h) and 13.2% for the sepsis management bundle (24 h) (Fig. 1).

### Comparison between cohorts

As displayed in Table 1, patients were divided into two cohorts: (1) elderly: 65–79 years and (2) very elderly: > 80 years. The two groups were similar in terms of number of organ failures (2.9 vs. 2.9, *p* = 0.88), invasive mechanical ventilation (59.3% vs. 62.0%, *p* = 0.428), and modified APACHE II score (17.2 vs. 16.7, *p* = 0.374).

**Table 1:** Comparison of baseline characteristics between elderly and very elderly patients

	Elderly (*n* = 1193) (80.1%)	Very elderly (*n* = 297) (19.9%)	Overall (*n* = 1490)	*p*
Age (years) mean (SD)	72.4 (4.1)	82.8 (2.7)	74.5 (5.6)	< 0.001 Ç
APACHE II modified score mean (SD)	17.2 (7.0)	16.7 (7.2)	17.1 (7.0)	0.374 Ç
Number of organs involved on admission mean (SD)	2.9 (1.4)	2.9 (1.4)	2.9 (1.4)	0.888 Ç
ICU LOS mean (SD)	14.5 (18.8)	11.5 (20.4)	13.9 (19.2)	0.014 Ç
Median	7.7	5.4	7.2	0.012
Median in survivors	7.8	7.5	7.4	0.7
Median in nonsurvivors	5.8	5.3	7.8	0.5
Mortality *N* (%)*	727 (48.8)	161 (54.2)	727 (48.8)	0.027*
Sex (male) *N* (%)*	719 (60.3)	157 (52.9)	876 (58.8)	0.020*
Admission category *N* (%)				
Medical	715 (60.2)	138 (46.6)	853 (57.2)	< 0.001*
Urgent surgery	361(30.4)	146 (49.3)	507 (34.0)	
Elective surgery	97 (8.2)	10 (3.4)	107 (7.2)	
Trauma	14 (1.2)	2 (0.7)	16 (1.1)	
Source of infection *N* (%)				
Pneumonia	391 (32.8)	68 (22.9)	459 (30.8)	< 0.001*
Peritonitis	396 (33.2)	135 (45.5)	531 (35.6)	
UTI	137 (11.5)	38 (12.8)	175 (11.7)	
SSTI	56 (4.7)	10 (3.4)	66 (4.4)	
Catheter-related bacteraemia	30 (2.5)	2 (0.7)	32 (2.1)	
Other	147 (12.3)	39 (13.1)	186 (12.5)	
Patient location at sepsis diagnosis *N* (%)				
ED	469 (39.3)	127 (42.8)	596 (40.0)	0.023*
Ward	547 (45.9)	144 (48.5)	691 (46.4)	
ICU	177 (14.8)	26 (8.8)	203 (13.6)	
Baseline acute organ dysfunction *N* (%)				
Cardiovascular	1023 (85.8)	245 (82.5)	1268(85.1)	0.158*
Pulmonary	250 (21.0)	62 (20.9)	312 (20.9)	0.976*
Renal	940 (78.8)	241 (81.1)	1181(79.3)	0.371*
Hepatic	191 (16.0)	50 (16.8)	241 (16.2)	0.730*
Thrombocytopenia	263 (22.0)	60 (20.2)	323 (21.7)	0.490*
Coagulopathy	415 (34.8)	104 (35.0)	519 (34.8)	0.941*
Sepsis resuscitation bundle *N* (%)^$^	102 (8.5)	14 (4.7)	116 (7.8)	0.027*
Sepsis management bundle *N* (%)^$^	165 (13.8)	32 (10.8)	197 (13.2)	0.164*

*APACHE II* acute physiology and chronic health evaluation II, *ICU* intensive care unit, *LOS* length of stay, *UTI* urinary tract infection, *SSTI* soft and skin tissue infections, *ED* emergency department

* *χ*2 tests; ^Ç^ Student’s *t* tests; ^$^ sepsis resuscitation bundle (six tasks that should begin immediately and be accomplished within the first 6 h of presentation) and the sepsis management bundle (four tasks that should begin immediately and be completed within 24 h of presentation)

Conversely, we found that very elderly patients had more intra-abdominal infections (46.5%, vs. 34.7%, *p* < 0.001) and, consequently, more urgent diagnostic surgical pathology (49.3% vs. 30.4%, *p* < 0.001) (data not shown in tables). Otherwise, the group of elderly had an increased number of medical admissions (60.2% vs. 46.6%, *p* < 0.001), and while the major source of infection was peritonitis, it was less frequent than in very elderly patients (33.2% vs. 45.5%, *p = *0.001). In addition, this group had more male patients (60.3% vs. 52.9%, *p = *0.02), an increased use of sepsis resuscitation bundles (8.5% vs 4.7%, *p = *0.027), a longer ICU LOS (14.5 ± 18.8 days vs. 11.5 ± 20.4 days, *p = *0.014). Further demographic and clinical characteristics of patients are shown in Table 1.

### Mortality and age in patients with sepsis and septic shock

The overall hospital mortality was 48.8% (*n* = 727) and was significantly higher in very elderly compared to elderly patients (54.2% vs. 47.4%; *p* = 0.02). The 28-day mortality was 39.6% (*n* = 590) and was significantly higher in very elderly compared to elderly patients (46.8% vs. 37.8%; *p* = 0.005). Risk factors for hospital mortality in elderly and very elderly patients are displayed in Table 2. According to the univariate analysis, independent variables significantly associated with hospital mortality were the following: modified APACHE II, number of organ failures, type of organ failure (respiratory failure, AKI, thrombocytopenia, and coagulopathy), source of infection, and patient location at sepsis diagnosis in the elderly cohort. On the other hand, in the very elderly cohort, the implementation of all measures of resuscitation within 6 h of diagnosis of sepsis and the location of the patient at the time of diagnosis were associated with mortality (Table 2).

**Table 2:** Risk factors for hospital mortality between elderly and very elderly patients

	Elderly *N* = 566	*p*	Very elderly *N* = 161	*p*
Age (years) mean (SD)	72.4 (4.1)	0.412Ç	83.0 (2.8)	0.329 Ç
APACHE II modified score mean (SD)	19.3 (7.2)	< 0.001Ç	18.4 (7.4)	0.012 Ç
Number of organs mean (SD)	3.2 (1.5)	< 0.001*	3.0 (1.5)	0.547*
Sex (male) *n* (%)	356 (49.5)	0.078*	86 (54.8)	0.835*
Admission category *n* (%)				
Medical	337 (47.1)	0.594*	66 (47.8)	0.056*
Urgent surgery	168 (46.5)		85 (58.2)	
Elective surgery	48 (49.5)		8 (80.0)	
Trauma	9 (64.3)		2 (100.0)	
Source of infection *n* (%)				
Pneumonia	223 (57.0)	< 0.001*	42 (61.8)	0.249*
Peritonitis	183 (45.7)		77 (57.0)	
UTI	36 (26.3)		14 (36.8)	
SSTI	19 (33.9)		4 (40.0)	
Catheter-related bacteraemia	12 (40.0)		1 (50.0)	
Other	72 (49.0)		21 (53.8)	
Patient location at sepsis diagnosis *n* (%)				
ED	188 (40.1)	< 0.001*	58 (45.7)	0.038*
Ward	271 (49.5)		87 (60.4)	
ICU	107 (60.5)		16 (61.5)	
Baseline acute organ dysfunction *n* (%)				
Cardiovascular	494 (48.3)	0.151*	133 (54.3)	0.954*
Pulmonary	145 (58.0)	< 0.001*	38 (61.3)	0.208*
Renal	472 (50.2)	< 0.001*	132 (54.8)	0.686*
Hepatic	106 (55.5)	0.015*	25 (50.0)	0.512*
Thrombocytopenia	150 (57.0)	< 0.001*	31 (51.7)	0.658*
Coagulopathy	229 (55.2)	< 0.001*	64 (61.5)	0.063*
Sepsis resuscitation bundle *N* (%)$	42 (41.2)	0.185*	45 (28.6)	0.049*
Sepsis management bundle *N* (%)$	72 (43.6)	0.291*	75 (46.9)	0.378*

*APACHE II* acute physiology and chronic health evaluation II, *ICU* intensive care unit, *LOS* length of stay, *UTI* urinary tract infection, *SSTI* soft and skin tissue infections, *ED* emergency department

*χ2 tests; ^Ç^Student’s *t* tests, ^$^sepsis resuscitation bundle (six tasks that should begin immediately and be accomplished within the first 6 h of presentation) and the sepsis management bundle (four tasks that should begin immediately and be completed within 24 h of presentation)

In the multivariable analysis, several risk factors remained independently associated with hospital mortality in the elderly cohort such as the modified APACHE II score (aOR 1.1 [95% CI 1.1–1.1], *p* ≤ .001), patient location at sepsis diagnosis (ward: aOR 1.5 [95% CI 1.1–2] *p* = 0.002, ICU: aOR 2.6 [95% CI 1.7–3.9] *p*  ≤ .001), AKI (aOR 1.4 [95% CI 1.0–2.0], *p* = 0.01), and thrombocytopenia (aOR 1.5, [95% CI 1.1–2.1], *p*  ≤ 0.01). In the very elderly cohort, age (aOR 1.1 [95% CI 1.1–1.2], *p* < 0.04), modified APACHE II score (aOR 1.1 [95% CI 1.1–1.1], *p*  ≤ 0.001), and the compliance in sepsis resuscitation bundle (6 h) according to the SSC recommendations (aOR 0.214 [95% CI 0.1–0.9], *p* < 0.05) were independent risk factors associated with hospital mortality (further details are shown in Table 3).

**Table 3:** Multivariate analysis for risk factors associated with hospital mortality between elderly and very elderly patients

	Elderly *N* = 566 aOR (CI 95%)	*p*	Very elderly *N* = 161 aOR (CI 95%)	*p*
Age (years)^a^	1.1 (0.9–1.0)	0.683	1.1 (1.0–1.2)	0.04
Sex (male)	0.8 (0.6–1.1)	0.231	1.1 (0.7–1.7)	0.710
APACHE II modified scoreb	1.1 (1.1–1.1)	0.000	1.1 (1.1–1.2)	< 0.001
ICU LOS	0.9 (0.989–1.003)	0.235	1.031 (1.0–1.0)	0.009
Patient location at sepsis diagnosisc				
Ward	1.5 (1.1–2.0)	0.002	1.5 (0.8–2.7)	0.130
ICU	2.6 (1.7–3.9)	0.000	0.6 (0.2–2.1)	0.507
ED (reference)	1.0		1.0	
Source of infectiond				
Peritonitis	0.6 (0.4–0.8)	0.005	0.9 (0.4–1.9)	0.856
UTI	0.3 (0.1–0.4)	0.000	0.3 (0.1–1.0)	0.059
SSTI	0.4 (0.2–0.8)	0.009	0.4 (0.1–2.1)	0.307
Catheter-related bacteremia	0.4 (0.2–1.0	0.055	0.9 (0.1–5.1)	0.243
Other	0.6 (0.4–1.4)	0.080	1.1 (0.4–2.7)	0.864
Pneumonia (reference)	1.0		1.0	
Baseline acute organ dysfunction				
Cardiovascular	1.0 (0.7–1.4)	0.886	0.6 (0.3–1.4)	0.316
Pulmonary	1.3 (0.9–1.8)	0.076	1.1 (0.5–2.3)	0.621
Renal	1.4 (1.0–2.0)	0.018	0.8 (0.4–1.7)	0.648
Hepatic	1.1 (0.8–1.7)	0.337	0.7 (0.3–1.6)	0.466
Thrombocytopenia	1.5 (1.1–2.1)	0.009	0.9 (0.4–2.1)	0.955
Coagulopathy	1.2 (0.9–1.7)	0.077	1.492 (0.8–2.6)	0.171
Sepsis resuscitation bundle *N* (%)^$^	0.8 (0.5–1.3)	0.437	0.214 (0.1–0.9)	0.031
Sepsis management bundle N (%)^$^	0.9 (0.6–1.3)	0.730	0.780 (0.3–1.8)	0.562

Elderly: 65–79 years of age and (2) very elderly ≥ 80 years of age

*aOR* adjusted odds ratio, *APACHE II* acute physiology and chronic health evaluation II, *ICU* intensive care unit; *LOS* length of stay, *UTI* urinary tract infection, *SSTI* soft and skin tissue infections

^a^Per age

^b^Per point

^c^Compared with the ED department

^d^Compared with pneumonia

^$^Sepsis resuscitation bundle (six tasks that should begin immediately and be accomplished within the first 6 h of presentation) and the sepsis management bundle (four tasks that should begin immediately and be completed within 24 h of presentation)

## Discussion

This prospective multicenter study found that mortality in old ages due to sepsis is high. Patients aged 80 or over had higher hospital mortality compared to patients between 65 and 79 years, and age represents an independent mortality-associated risk factor in this very elderly cohort while it is not in the younger population cohort. Interestingly, there are elements identified that would improve hospital survival, in very elderly patients, such as the resuscitation bundle provided in the first hours of hospital admission. Despite the high mortality associated with this very elderly population (54.2%), appropriate and prompt medical management in ICU ultimately impacts in patients’ survival.

According to the results of the 2017 revision of the world population prospects, Europe is today facing unprecedented demographic change: 25% of the population is already aged 60 years or over and that proportion is projected to reach 35% in 2050, while the number of persons aged 80 or over is going to triple by 2050 [20]. Therefore, the mean age of patients admitted to the hospital and ICU has also increased. Almost two out of three patients admitted to our study for sepsis were “patients aged 65 years or older.” Martin et al. [10] reported a similar percentage, showing that age is associated with the development of sepsis and with the outcome. Angus et al. reported a steadily increased mortality with patients’ age, with a significant peak of almost 40% in patients older than 85 years [8]. In a historical cohort study comparing middle-aged (45–64 years), old (65–74 years), and very old ICU patients (> 75 years), Blot et al. [21] found that mortality rates increased with age: 42.9%, 49.1%, and 56.0% for middle-aged, old, and very old patients, respectively.

In our study, the very elderly cohort had a lower implementation of resuscitation bundles after sepsis diagnosis. Interestingly, this finding is in agreement with previous studies, showing that age is an independent factor for the limitation of treatment in critically ill patients. Does it reflect a poor encouragement from the attending physician to change patients’ outcome? Therefore, the older is the patient; the lower is the implementation of therapy. However, and despite of this common clinical sense, prompt therapy provided within the first 6 h of resuscitation was associated with a reduction in hospital mortality in this subgroup of patients.

In our study, as age can contribute up to 6 points, the level of severity at admission was assessed by a modified APACHE II score, in which the impact of age on the APACHE score was eliminated. The overall mortality of our study is 48.8%. This is high if compared to the VIP1 study, a prospective multinational study involving 5132 very old intensive care patients with a median age of 84 years from 311 ICUs. In the VIP 1 study, the 30-d mortality is, respectively, 43% for the subgroup of acute medical admission (*n* = 3245 patients admitted) and 26.4% for the acute surgical admission (*n* = 382 patients) and 47% for trauma admission (*n* = 228 patients). However, the reason for admission was not only sepsis/septic shock and, according to the multivariate analysis, but only the acute admissions have the strongest impact on survival, while age has a smaller impact. The strength of the study is that is focused on the septic subgroup (sepsis and septic shock): 83% of them with septic shock and with a median LOS in ICU of 13.9 days, wherein the VIP 1 study the median ICU LOS was much shorter (2.3 days) and included more than 3000 acute patients. In addition, our study is in line with other studies about elderly patients with sepsis and septic shock, where the mortality rates are around 50–60% [2, 10, 12]. We have to acknowledge that in our manuscript, we incorporated bundles before the new update of SSC guidelines, as the largest reductions in mortality have been associated with early identification of sepsis, initiation of a 3-h care bundle, and prescription and administration of broad spectrum antibiotics within the first hour. Despite current extraordinary investment in sepsis implementation awareness and management, there is still a lack of progress in mortality reduction in sepsis treatment that underscores the variability in patients with sepsis.

Life-sustaining treatment (LST) limitations before and during ICU admission related to comorbidities, ventilator support time, noninvasive mechanical ventilation, quality of life, frailty, and/or functional status were not recorded and are major limitations in our study. Additionally, the lack of a well-known organ failure score such as the sequential organ failure assessment (SOFA) score could be used for comparison of results with other similar studies. Only 20% of patients admitted to ICU for sepsis or cardiovascular failure were 80 years or over and a minority (0.4%) were 90 or over. However, a French prospective, observational multicenter cohort study about the admission of patients older than 80 years to ICU showed that not only emergency physicians were unlikely to refer octogenarian patients, but also intensivists were reluctant to admit them despite the presence of criteria indicating an appropriate admission [22]. Therefore, this might explain why the number of patients included in the very elderly cohort was low, and might justify the inequality of sample size between the two cohorts (elderly and very elderly). However, this selection bias would be minimized since there were no significant differences between the groups in any of the weighted variables: modified APACHE II score and baseline acute organ dysfunction, number and incidence of organ failures. Considering the differences between the two groups in sample size, in clinical characteristics and in the univariate analysis of mortality, we performed a multivariate analysis for each cohort, adjusted to the same logistic regression model.

The initial treatment strategy followed the treatments recommended by the SSC guidelines [19] available at the time of the study and now outdated. However, the important message is that prompt management in the first hours of sepsis is beneficial also in the patients that are usually considered for a less intense treatment. This is in line with recent studies that are proving that the intensity of care in elderly patients is increasing with beneficial effects in these patients [15, 23, 24]. Similarly, we should study the long-term survival and the quality of life at 6 months after discharge from the hospital, in order to create predictive models to guide the decision-making for admitting elderly and very elderly patients in ICU. Another important concept that should be further investigated as a predictor of outcome in the elderly patients is the frailty that measures the susceptibility from the age-associated decline in reserve and function in a wide range of physiological systems [25]. Recently, frailty was associated with an increased risk of mortality in critically ill patients older than 80 years [15] and generally in the elderly patients [26].

## Conclusion

This prospective multicenter study about elderly critically ill patients with sepsis shows that patients aged 80 or over had higher hospital mortality compared to patients between 65 and 79 years. Age was found to be an independent risk factor only in the very elderly group, and prompt therapy provided within the first 6 h of resuscitation was associated with a reduction in hospital mortality in the very elderly patients.
