# Correction to: Risk factors for mortality in elderly and very elderly critically ill patients with sepsis: a prospective, observational, multicenter cohort study

**DOI:** 10.1186/s13613-019-0508-9

**Published:** 2019-03-13

**Authors:** Ignacio Martin-Loeches, Maria Consuelo Guia, Maria Sole Vallecoccia, David Suarez, Mercedes Ibarz, Marian Irazabal, Ricard Ferrer, Antonio Artigas

**Affiliations:** 1 Multidisciplinary Intensive Care Research Organization (MICRO), St James’s Hospital/Trinity College Dublin TCD, James’s St, Ushers, Dublin, D03 VX82 Ireland; 2 Pulmonary Intensive Care Unit, Respiratory Institute, Hospital Clinic of Barcelona, IDIBAPS, Barcelona, Spain; 3 University of Barcelona, Barcelona, Spain; 4 Centro de Investigación Biomédica en Red-Enfermedades Respiratorias (CIBERES CB06/06/0028), Barcelona, Spain; 5 Critical care Center, Corporacion Sanitaria Universitaria Parc Tauli, CIBER Enfermedades Respiratorias, Autonomous University of Barcelona, Sabadell, Spain; 6 Department of Intensive Care and Anaesthesiology, Università Cattolica del Sacro Cuore - Fondazione Policlinico Universitario A.Gemelli, Rome, Italy; 7 Servicio de Medicina Intensiva, Hospitales Universitarios Sagrado Corazon y General de Cataluña, Barcelona, Spain; 8 Intensive Care Department, Vall d’Hebron University Hospital, Shock Organ Dysfunction and Resuscitation Research Group, Vall d’ Hebron Research Institute, Passeig de la Vall d’Hebron, 119-129, 08035 Barcelona, Spain

## Correction to: Ann. Intensive Care (2019) 9:26 10.1186/s13613-019-0495-x

Following the publication of the original article [1], the author reported two mistakes in Fig. 1:

1. The note “1490 patients with ≥ 65 years of age” was missing;

2. Elderly (*n* = 119) (80.1%) should be replaced with Elderly (*n* = 1193) (80.1%).

It has been corrected in the original article as well. The publisher apologizes for any inconvenience caused by this error.
